# Accelerating global efforts to prevent women’s cancers^☆^

**DOI:** 10.1016/j.mmr.2026.100062

**Published:** 2026-08-12

**Authors:** Leeya F. Pinder

**Affiliations:** a Department of Obstetrics and Gynecology, Division of Gynecologic Oncology, University of Cincinnati, Cincinnati, OH 45219, USA; b University of Cincinnati Cancer Center, College of Medicine, University of Cincinnati, Cincinnati, OH 45219, USA

**Keywords:** Women’s cancer, Breast cancer, Cervical cancer, Ovarian cancer, Uterine cancer, Disease burden, Group of Twenty (G20)

Women’s cancers, including breast, cervical, ovarian, and uterine (endometrial) cancers, collectively impose a profound and inequitably distributed global burden [1], [2]. Breast cancer is the most common female malignancy worldwide, while cervical cancer remains the leading cause of death from gynecological cancers in low- and middle-income countries (LMICs) where late-stage diagnosis predominates [2]. Ovarian and uterine cancers contribute a substantial and rising share to this burden, with increasing incidence linked to rising obesity prevalence [1]. Against this backdrop, longitudinal epidemiological assessments are essential to inform targeted interventions and resource allocation. The study by Li *et al*. [3] provides the most comprehensive update to date on incidence, prevalence, mortality, disability-adjusted life years (DALYs), quality of care index (QCI), and survival for these four cancers across 98 locations of the Group of Twenty (G20) from 1990 to 2023. This commentary contextualizes Li *et al*. [3] by examining the principal drivers of the changing burden, identifying persistent challenges, and offering evidence-based public health recommendations.

The observed decline in age-standardized DALY rates of women’s cancers across the G20 locations, as documented by Li *et al*. [3], reflects the effectiveness of decade-long investments in early detection and immunization [2]. Screening programs and human papillomavirus (HPV) vaccination have been instrumental in reducing the burden of breast and cervical cancers in high-income settings. Sweden introduced the world’s first nationwide, population-based breast cancer screening program in 1986, inviting women aged 40–74 years to biennial mammography with an average participation rate of approximately 70% [4]. Over three decades of follow-up, a 40% increase in 25-year cumulative breast cancer mortality among non-participants was observed [4], establishing the strongest longitudinal evidence base for organized screening globally. In the United Kingdom, women aged 25–50 years and 50–64 years are invited for screening every three and five years, respectively. From 2008, the national HPV vaccination program has been introduced and has been gradually covering girls aged 9–13 years. Each year, up to 3900 cases of cervical cancer are prevented among around 3.5 million eligible women [5]. In their analysis, Li *et al*. [3] highlight relatively lower age-standardized DALY rates of breast cancer in Sweden (402.85/100,000) and cervical cancer in the United Kingdom (90.80/100,000) in 2023. These successes share a common architecture, high population coverage, institutional delivery platforms, and sustained investment.

Li *et al*. [3] presented increasing trends of QCI, 5-year relative survival, and prevalence of women’s cancers, which may be associated with therapeutic advances and demographic transition. Therapeutic advances, including surgery, radiation therapy, and cancer medicines (systemic therapy), significantly impact the cure or survival of women’s cancer [6]. For instance, the introduction of human epidermal growth factor receptor 2-targeted therapy and immune checkpoint inhibitors has shifted 5-year breast cancer survival above 90% in high-income settings [6]. Despite these gains, Li *et al.* [3] rightly identify an upward trend in absolute numbers of cases and deaths. Population growth and aging mean that declining per-capita rates still translate into more total cases, placing increasing demands on health-system capacity [1], [2].

The fertility-cancer nexus represents a critical and often underappreciated determinant of women’s cancer risk. A particularly important finding of Li *et al*. [3] is the association between declining total fertility rates (TFRs) and increased DALYs across all four cancer types. The mechanism of the fertility-cancer nexus is biological; for instance, each full-term pregnancy reduces cumulative lifetime estrogen exposure through gestation and lactation, conferring protection against breast, ovarian, and uterine cancers [6], [7]. As TFRs fall globally, this hormonal protection diminishes at the population level, structurally amplifying cancer risk in ways that clinical interventions alone cannot fully counteract.

Several persistent challenges continue to impede equitable progress in women’s cancer control. First, geographic and racial inequities persist. African Union countries bear the highest DALY rates and lowest survival rates, compounded by high HPV prevalence and low screening rates [2]. Increasing global health equity requires deliberate workforce diversification and community-embedded screening models rather than passive expansion of existing services. Second, ovarian cancer lacks an evidence-based national screening program, given the absence of mortality benefit in major randomized trials. The United Kingdom Collaborative Trial of Ovarian Cancer Screening enrolled 202,638 women with a median follow-up of 16 years and demonstrated that neither annual cancer antigen 125 testing nor transvaginal ultrasound reduced ovarian cancer mortality [8]. Consequently, approximately 54% of ovarian cancers are diagnosed at an advanced stage, when the 5-year survival rate falls around 30% [6]. To address this challenge, actions must pivot from population screening toward risk-stratified early detection and investing in multi-cancer early detection liquid biopsy platforms that may eventually capture ovarian malignancies at a curable stage. Third, reproductive health and cancer prevention programs remain siloed, forfeiting high-value prevention opportunities at antenatal, postpartum, and family planning contacts. Separately, HPV vaccine coverage stagnates well below the World Health Organization’s (WHO’s) 90% target in many countries [6], due to structural barriers in LMICs and social misinformation in middle-income countries. Advocate for oncology-receptive reproductive health platforms where antenatal, postpartum, contraceptive, or menopausal triggers age-appropriate cancer prevention interventions.

Addressing the burden of women’s cancers requires multifaceted strategies tailored to regional contexts. For cervical cancer, accelerating HPV vaccination toward the WHO’s 90–70–90 targets through single-dose schedules, now proven to offer durable protection at lower cost [9], and integrating programs into the school health infrastructure remains critical. Shifting to HPV DNA-based primary screening, recommended by WHO’s 2021 guidelines as the preferred primary cervical screening method [10], with self-collection can dramatically improve uptake among never-screened women. However, it is important to recognize that this robust prevention paradigm for cervical cancer stands in stark contrast to the other three women’s cancers: breast, ovarian, and uterine cancers, which currently lack validated population-level screening or prevention modalities. Furthermore, integrating cancer prevention into reproductive health platforms is supported by the fertility-cancer nexus that Li *et al.* [3] demonstrate. Antenatal, postpartum, and family planning contacts should routinely include HPV vaccination catch-up, cervical cancer screening, clinical breast examination, and breastfeeding counseling [2], [7]. Rwanda’s integration of HPV vaccination into its national immunization program achieved over 90% coverage and provides a replicable model for high-burden, resource-limited settings [6]. Finally, investing in cancer surveillance and global financing is essential. LMIC governments should establish sentinel registry networks using standardized data collection systems. Development finance institutions and national governments must treat women’s cancer prevention as a core health financing priority rather than a discretionary program.

In brief, the findings of Li *et al.* [3] present a dual reality, with meaningful progress in age-standardized rates where health systems have invested, yet persistent and widening inequity where they have not. The tools for transformative change already exist. Translating them into universal impact requires policy prioritization, resource mobilization, integrated service delivery, and financing commensurate with the scale of the burden, thereby accelerating the convergence of women’s cancer outcomes across geographies and generations.

## Abbreviations

DALYs: Disability-adjusted life years

G20: Group of Twenty

HPV: Human papillomavirus

LMICs: Low- and middle-income countries

QCI: Quality of care index

TFRs: Total fertility rates

WHO: World Health Organization

## Declarations

### Ethics approval and consent to participate

Not applicable.

## Author’s contributions

LFP was responsible for the design, conception, and drafting of the manuscript. The author read and approved the final manuscript.

## Funding

Not applicable.

## Competing interests

The author declares that there is no competing interests.

## Data Availability

Not applicable.
